# An RNA methylation code to regulate protein translation and cell fate

**DOI:** 10.1111/cpr.13224

**Published:** 2022-03-30

**Authors:** Dan Song, Ng Shyh‐Chang

**Affiliations:** ^1^ State Key Laboratory of Stem Cell and Reproductive Biology Chinese Academy of Sciences Beijing China; ^2^ Institute for Stem Cell and Regeneration Chinese Academy of Sciences Beijing China; ^3^ University of Chinese Academy of Sciences Beijing China; ^4^ Beijing Institute for Stem Cell and Regenerative Medicine Beijing China

Dear editor,

In the past few decades, a flurry of studies has revealed the importance of RNA methylation. These modifications are present in various types of RNAs and collaborate with “writers”, “erasers” and “readers” to influence RNA metabolism and regulate cell differentiation and transformation. In particular, protein synthesis can be directly influenced by RNA codon‐recognition and structure‐based factors or indirectly by RNA methylation reader proteins. Here, we briefly describe the important role of RNA methylation in tumorigenesis and stem cell differentiation, and focus on how major RNA methylations alter translation rates via ribosomal activity or codon usage. These regulatory mechanisms potentially regulate protein diversity through amino acid polymorphisms. With the improvement of single‐base modification and amino acid sequencing technologies, the complex roles of RNA modifications in ribosomal translation and cell fate determination are being revealed.

## INTRODUCTION

In recent decades, with the development of next generation sequencing (NGS), various detection methods for RNA modifications have been gradually developed and refined. On this basis, a basic understanding of the types, abundance and distribution patterns of RNA modifications has been obtained.^1^, ^2^ To date, more than 170 RNA modifications have been identified in various types of RNAs, and interest in the biological functions of these modifications has led to the establishment and development of epigenetics and epitranscriptomics.^3^, ^4^, ^5^

Among the diverse types of RNA modifications, the main ones that have been studied are N6‐methyladenosine (m^6^A), 5‐methylcytosine (m^5^C), N1‐methyladenosine (m^1^A), and N7‐methyladenosine (m^7^G). How do cells generate these modifications in response to internal and external metabolism during cell fate determination, e.g., differentiation and transformation? What are the implications of these modifications in the process of genetic information transmission?

## m^6^A IN CELL FATE DETERMINATION

m^6^A is a reversible modification present on the adenine residue of many RNAs. A variety of methyl‐group transferases, demethylases and reader proteins work together to regulate the dynamics of this modification, such as METTL3/14, FTO, YTHDF1‐3 etc.^6^, ^7^ Combined with these protein factors, m^6^A is involved in multiple post‐transcriptional processes, including splicing,^8^ processing,^9^ translocation,^10^ RNA stability^11^ and translation efficiency.^12^

Although m^6^A modifications have been studied in a variety of biological processes, less attention has been paid to the role of m^6^A in regulating codon‐specific translation dynamics. The m^6^A‐modified codons in mRNA may reduce the accuracy of codon reading by tRNAs and peptide release factors, and m^6^A‐U pairings are possibly less stable relative to A‐U pairings.^13^, ^14^ The m^6^A located within the coding region (CDS) directly leads to ribosome pausing. Conversely, CDS m^6^A binding to YTHDC2 facilitates mRNA secondary structure opening and increases translation efficiency.^15^ Additionally, when m^6^A is present at the tRNA's anticodon stem and loop (ASL) domain, the N6‐adenosine electron clouds and dynamic structure of the ASL can be altered to produce codon wobble, which affects translation fidelity and causes protein noise.^16^ In the case of ribosomal rRNAs, 18S m^6^A_1832_ and 28S m^6^A_4220_ are known to stabilize ribosome structure and subunit assembly.^17^, ^18^, ^19^ These studies suggest that m^6^A regulates codon diversity and protein translation, which alters the transmission rate of genetic information. This could be another reason why this modification plays an important role in regulating embryonic development,^20^, ^21^ stem cell differentiation,^22^, ^23^ viral replication^24^, ^25^, ^26^ and tumour progression.^27^, ^28^, ^29^

## m^5^C IN CELL FATE DETERMINATION

m^5^C is a class of cytosine methylation in many RNAs. It is mainly catalyzed by NOL1/NOP2/sun domain family proteins (NSUNs) or DNA methyltransferase 2 (DNMT2).^2^, ^30^, ^31^ Studies have shown that m^5^C has an important role in regulating cell fate and development. For example, during the maternal‐to‐zygotic transition (MZT) in zebrafish embryogenesis, m^5^C‐modified maternal mRNAs are more stabilized by recruiting Y‐box binding protein 1 (YBX 1) and poly (A) binding protein cytoplasmic 1a (Pabpc1a).^32^ In epidermal stem cells, NSUN2‐mediated m^5^C protects tRNA from cleavage into non‐coding 5′tRNA fragments, thereby affecting global protein synthesis patterns.^33^ There is also evidence that m^5^C is present in the wobble position of tRNA anticodons, thereby regulating the translation efficiency of leucine and proline, with resultant effects on the oxidative stress response in yeast and the heat stress response in *Caenorhabditis elegans*.^34^, ^35^ Based on an analysis of in vitro translation efficiency, m^5^C at any of the codon positions resulted in a 40% decrease in protein production.^36^

The emerging role of m^5^C in many cancers' progression has been widely studied. The m^5^C methyltransferases (including NSUN2 and DNMT2) are highly expressed in a variety of tumour tissues, causing multiple oncogene mRNAs to be hypermethylated and stabilized.^37^, ^38^, ^39^ For example, NSUN2 increases the m^5^C content of heparin binding growth factor's (HDGF) mRNA in bladder cancer, recruiting YBX1 and ELAV‐like RNA binding protein 1 (ELAVL1) to maintain the high expression of HDGF mRNA, thereby promoting the proliferation and invasion of bladder cancer cells.^40^

## m^1^A AND m^7^G IN CELL FATE DETERMINATION

Studies have shown that the level of m^1^A modification is about one‐tenth of that of m^6^A, and it is mainly distributed in tRNA, rRNA, 5′UTR of mRNA and mitochondrial DNA‐encoded transcripts.^41^, ^42^, ^43^, ^44^, ^45^ Similar to m^6^A, m^1^A has effects on the tertiary conformation of tRNA and rRNA, and regulates overall translation efficiency. For instance, AlkB homologue 1 (ALKBH1) was identified as a tRNA demethylase, which mediates the demethylation of m^1^A, resulting in attenuated translation initiation and reduced total protein synthesis.^46^ In addition, 26S rRNA m^1^A_674_ was found to be catalyzed by Rram‐1 in *Caenorhabditis elegans*,^47^ and 25S rRNA m^1^A_645_ was found to be catalyzed by Rrp8 in yeast,^48^ with positive effects on ribosomal subunit assembly. It has been shown that m^1^A drives cancer cell proliferation and promotes the development of gastrointestinal cancer,^49^ bladder uroepithelial cancer^50^ and hepatocellular carcinoma,^51^ but its role in stem cell differentiation remains unclear.

The m^7^G was initially identified as a signature modification in the mRNA 5′ cap structure. Subsequently, m^7^G was also found in rRNA, tRNA and internal mRNA, with an especially high enrichment in the 5′ UTR region and AG‐rich regions.^52^, ^53^ Studies have shown that METTL1 catalyzes m^7^G in pri‐miRNA, and this methylation promotes miRNA processing by antagonizing G‐quadruplex structures, thereby increasing let‐7e‐5p miRNA.^54^ In mESCs, the Mettl1/Wdr4 complex regulates the tRNA m^7^G methylome, thereby regulating global mRNA translation, stem cell self‐renewal and differentiation.^55^

## CONCLUSIONS AND PERSPECTIVES

In recent decades, with the establishment of various new techniques to detect rare RNA modifications, researchers have gradually revealed the roles of m^6^A, m^5^C, m^1^A and m^7^G in many biological processes and diseases. In collaboration with writers, readers and erasers, RNA methylation codes affect the tertiary conformation, processing and stability of RNA, with multiple effects on translation initiation, elongation and termination. In fact, due to the low abundance of these RNA modifications (except for m^6^A perhaps), more sensitive detection techniques are still needed, and the roles of RNA methylation in cell differentiation and transformation remain to be further explored. On the other hand, because the influence of RNA methylation on the specificity of codon‐anticodon pairing has direct relevance to Crick's wobble hypothesis, the resultant amino acid polymorphisms in protein distributions may be a very interesting biological phenomenon that deserves further studies.

## CONFLICT OF INTEREST

All authors declare that they have no conflict of interest.

## AUTHOR CONTRIBUTIONS

Dan Song and Ng Shyh‐Chang designed and wrote the manuscript.

## Data Availability

The authors declare that all the data supporting the findings of this study are available within the article and from the corresponding authors upon reasonable request.
